# Nirsevimab in the New Era of RSV Prevention: Current Evidence, Emerging Challenges, and Future Perspectives

**DOI:** 10.3390/medicina62101858

**Published:** 2026-09-25

**Authors:** Marina Vasou, Rozeta Sokou, Georgios Kafalidis, Andreas G. Tsantes, Rafaela Anna Moutsopoulou, Fotios Grigoropoulos, Eleni Katsianou, Nikoleta-Aikaterini Xixi, Elli-Panagiota Magklara, Konstantina Leontari, Zoi Iliodromiti, Theodora Boutsikou, Nicoletta Iacovidou

**Affiliations:** 1 Neonatal Department, National and Kapodistrian University of Athens, Aretaieio Hospital, 11528 Athens, Greece; marinavaso0@gmail.com (M.V.); gkafalidis@gmail.com (G.K.); ekatsianou@med.uoa.gr (E.K.); ellimaglara99@gmail.com (E.-P.M.);; 2 Department of Microbiology, Saint Savvas Oncology Hospital, 11522 Athens, Greece; atsantes@med.oua.gr

**Keywords:** Nirsevimab, respiratory syncytial virus, infants, newborns, immunoprophylaxis, monoclonal antibodies

## Abstract

Respiratory syncytial virus (RSV) is the leading cause of lower respiratory tract infection (LRTI) and hospitalization in infants and young children worldwide, with the highest disease burden occurring in otherwise healthy term infants during the first 6 months of life. The recent introduction of long-acting monoclonal antibodies and maternal RSV vaccination has fundamentally changed preventive strategies against severe RSV disease. Evidence from randomized clinical trials and large real-world studies consistently demonstrates that a single dose of Nirsevimab provides season-long protection against RSV-associated LRTI and reduces hospitalization rate across diverse infant populations, while maintaining an excellent safety profile. Emerging data also support its effectiveness in routine clinical practice, although important uncertainties remain regarding long-term epidemiological effects, viral evolution, resistance, comparative effectiveness with maternal immunization, and optimal implementation across different healthcare settings. Current evidence supports Nirsevimab as a major advance in infant RSV prevention. However, continued surveillance, comparative effectiveness studies, and equitable global implementation will be essential to maximize its long-term public health impact. This narrative review summarizes current evidence on Nirsevimab, with emphasis on its clinical efficacy, safety, real-world effectiveness, resistance, implementation challenges, cost-effectiveness, and its role alongside maternal RSV vaccination.

## 1. Introduction

Respiratory syncytial virus (RSV) is a leading cause of acute respiratory tract infection worldwide and remains a major contributor to pediatric morbidity and mortality among infants and young children. The incidence of RSV infection is highest during the first two years of life, with an estimated rate of 5.2 cases per 1000 live births, and increases markedly to 25.9 cases per 1000 live births in infants younger than one month of age [1]. Globally, RSV-associated acute LRTIs are estimated to cause approximately 33 million episodes annually in children younger than 5 years, resulting in more than 3 million hospitalizations and 59.600 in-hospital deaths. Notably, infants younger than 6 months account for approximately 1.4 million hospitalizations and 27.300 in-hospital deaths [2]. These figures emphasize that, although RSV infection is widespread across early childhood, its clinical impact is disproportionately concentrated in the first 6 months of life, making this period a critical target for preventive interventions. Although most infections are self-limiting, RSV is responsible for a substantial number of outpatient visits, hospitalizations, intensive care unit admissions, and deaths, particularly in young infants and those with underlying medical conditions. The most severe manifestations of RSV disease occur in the first six months of life, accounting for approximately 50–90% of pediatric hospitalizations for bronchiolitis and 5–40% of hospitalizations for pneumonia [2,3,4,5].

For more than two decades, RSV prevention relied almost exclusively on palivizumab, a monoclonal antibody administered monthly throughout the RSV season. Owing to its high cost, repeated dosing requirements, and associated logistical challenges, its use was largely restricted to infants with the highest risk of severe disease, including those born prematurely, with chronic lung disease, or with hemodynamically significant congenital heart disease. Consequently, the majority of healthy infants, who account for a considerable proportion of RSV-related hospitalizations, remained without an effective preventive intervention [5,6,7,8].

Recent advances in RSV immunoprophylaxis have substantially broadened preventive options and shifted the focus toward population-based protection. Two complementary strategies are currently available: maternal immunization and long-acting monoclonal antibodies for the offspring.

Maternal vaccination with the RSV prefusion F protein vaccine (Abrysvo) administered between 32 and 36 weeks of gestation provides passive protection through the transplacental transfer of maternal antibodies [7]. Immunologically, this strategy relies on active maternal immunization to induce maternal RSV-specific antibody responses, while newborn protection is passive and results from transplacental transfer of maternal antibodies. However, several factors influence transplacental antibody transfer, including gestational age, placental function, and maternal health status In a prospective cohort study, maternal vaccination 2–3 or 3–4 weeks before delivery was associated with significantly lower cord-to-maternal antibody transfer than vaccination more than 5 weeks before delivery. These findings suggest that the timing of maternal immunization relative to delivery may influence the magnitude of passive protection achieved at birth [9]. As a result, both the effectiveness and duration of protection may be reduced, since maternally derived antibodies decline over time and may not provide coverage for the entire RSV season, particularly in tropical and subtropical regions [10].

The second major prevention strategy involves directly administering extended-half-life monoclonal antibodies (Nirsevimab) to infants. Unlike maternal vaccination, Nirsevimab (commercial name Beyfortus) provides direct passive immunoprophylaxis through the administration of a preformed RSV-specific monoclonal antibody, without inducing an active immune response or immunological memory in the infant. Compared with palivizumab, which requires monthly administration and is limited to high-risk populations, Nirsevimab is designed for single-dose administration that protects throughout the RSV season. This approach represents a paradigm shift from risk-stratified prophylaxis toward universal RSV prevention, applicable to all infants entering their first respiratory syncytial virus season. Data from randomized clinical trials and supported by more recent real-world evidence consistently indicate that Nirsevimab significantly reduces the risk of severe RSV disease and maintains a favorable safety profile [3,6,7,11,12].

Despite the promising evidence supporting Nirsevimab, several important issues remain to be clarified. These include the duration of protection beyond a single RSV season, the potential effects of widespread implementation on RSV epidemiology and viral evolution, the emergence of variants with reduced susceptibility to Nirsevimab, and the optimal integration of Nirsevimab within current RSV prevention strategies, particularly in the context of maternal RSV immunization. Expanding evidence from real-world studies following the widespread implementation of Nirsevimab, alongside emerging findings on viral escape, maternal vaccination, and implementation challenges, has created a rapidly evolving evidence base. This underscores the need for an updated and comprehensive evaluation of the role of Nirsevimab in the contemporary RSV prevention landscape. Therefore, this review aims to provide a critical overview of the current evidence on the development, mechanism of action, clinical efficacy, safety, and real-world use of Nirsevimab; examine its role in contemporary RSV prevention; and identify key challenges and knowledge gaps that require further investigation.

## 2. Materials and Methods

A structured literature search was performed using the electronic databases PubMed and Scopus, and Google Scholar was used as a supplementary source to identify relevant publications. The search strategy combined Medical Subject Headings (MeSH) and free-text terms, including “Nirsevimab,” “respiratory syncytial virus,” “RSV,” “infants,” “newborns,” “RSV prevention,” “maternal RSV vaccination,” “immunoprophylaxis,” and “monoclonal antibodies.” Eligible publications included randomized controlled trials, observational studies, systematic reviews, meta-analyses, clinical practice guidelines, regulatory documents, and other original research published in English. Conference abstracts without available full texts, duplicate publications, and studies that did not provide sufficient methodological or clinical information relevant to the scope of the review were excluded. In addition, the reference lists of eligible articles and relevant reviews were manually screened to identify additional publications. The retrieved literature was critically synthesized with emphasis on clinical efficacy, safety, real-world effectiveness, implementation strategies, resistance, cost-effectiveness, maternal RSV vaccination, public health implications of Nirsevimab, and future perspectives. This review emphasized recent high-quality clinical evidence, including landmark randomized trials, large observational studies, and current international recommendations. As this study was designed as a narrative review, no formal assessment of methodological quality or risk of bias was performed.

## 3. Clinical Development and Regulatory Approval

The development of Nirsevimab represents the culmination of decades of research aimed at overcoming the limitations of existing strategies for the prevention of respiratory syncytial virus (RSV) disease. Progress in understanding RSV pathogenesis, particularly the structural biology of the viral fusion (F) protein, and the identification of highly vulnerable antigenic epitopes, with advances in monoclonal antibody engineering, enabled the development of next-generation immunoprophylactic agents capable of providing prolonged protection following a single administration.

For more than two decades, palivizumab remained the only licensed immunoprophylactic agent against RSV. The development of palivizumab was based on the progressive understanding of the RSV F glycoprotein as a key target for virus neutralization. Approved by the U.S. Food and Drug Administration (FDA) in 1998, this humanized monoclonal antibody targeted the RSV F protein and was administered as monthly intramuscular injections (15 mg/kg) throughout the RSV season. Its use was restricted to infants at high risk of severe RSV disease, including those born prematurely and those with chronic lung or congenital heart disease [3,6,7].

Clinical trials demonstrated that palivizumab reduced RSV-related hospitalizations by approximately 55% overall (from 10.6% in the placebo group to 4.8% in treated infants), with greater efficacy in premature infants without bronchopulmonary dysplasia (78% reduction) than in infants with bronchopulmonary dysplasia (39% reduction) [13,14]. The benefit was also reflected in a reduction in total RSV hospital days, days requiring supplemental oxygen, and PICU admission [14]. Despite its demonstrated efficacy, the limitations outlined above restricted the widespread use of palivizumab, leaving the majority of healthy infants without an effective preventive intervention.

Recognizing the need for a more practical, broadly applicable approach to RSV prevention, Nirsevimab was developed as a long-acting monoclonal antibody engineered to provide season-long protection after a single intramuscular dose. Its efficacy and safety were subsequently established through a comprehensive clinical development program conducted across diverse infant populations, ultimately leading to regulatory approval by international health authorities and its integration into contemporary RSV prevention strategies. The main characteristics and key differences between Nirsevimab and palivizumab for RSV prevention are summarized in Table 1.

### 3.1. Molecular Design and Mechanism of Action of Nirsevimab

Advances in the understanding of RSV molecular biology were fundamental for the development of Nirsevimab. RSV is an enveloped, single-stranded RNA virus of the genus *Orthopneumovirus*, comprising two major antigenic subtypes, RSV-A and RSV-B, which commonly co-circulate during seasonal outbreaks [15,16]. Among the viral surface proteins, the fusion (F) glycoprotein represents a major target for neutralizing antibodies, due to its markedly greater genetic and antigenic conservation across RSV subtypes compared with the attachment (G) glycoprotein [17]. A major advance in RSV prevention was achieved with the discovery of the prefusion conformation of the F protein and the identification of highly conserved neutralizing epitopes, particularly the antigenic site Ø, which set the foundation for the rational design of next-generation monoclonal antibodies and vaccines [17].

Nirsevimab was specifically engineered to overcome key limitations of earlier anti-RSV monoclonal antibodies such as palivizumab through two major structural modifications. It is a fully human recombinant IgG1κ monoclonal antibody derived from MEDI8897 that specifically targets antigenic site Ø (zero) on the prefusion conformation of the RSV fusion (F) glycoprotein. Structural and immunological studies identified site Ø as one of the most conserved and immunologically vulnerable epitopes on the prefusion F protein, making it an ideal target for antibody-mediated neutralization. Unlike palivizumab, which binds antigenic site II present on both prefusion and postfusion F proteins, Nirsevimab binds exclusively to site Ø with substantially greater affinity and neutralizing potency. By engaging both the F1 and F2 subunits, Nirsevimab stabilizes the prefusion structure, enhancing neutralization potency [6,11,18].

The selection of antigenic site Ø as the target epitope translated into potent antiviral activity in preclinical studies. Nirsevimab demonstrated strong neutralizing activity against both RSV-A and RSV-B isolates, and exhibited markedly greater in vitro potency compared with palivizumab [12]. These observations were further supported in preclinical models of RSV infection, where Nirsevimab demonstrated greater suppression of viral replication in the upper and lower respiratory tracts, providing the biological rationale for subsequent clinical development [19].

A second major innovation in Nirsevimab design was incorporating the YTE (M252Y/S254T/T256E) triple amino acid substitution within the CH2 domain of the Fc region. The Fc region consists of two identical heavy chain constant region domains (CH2 and CH3) that dimerize to form the characteristic “stem” of the Y-shaped antibody molecule. The CH2 domain, where the YTE substitutions are located (specifically at positions 252, 254, and 256 according to the EU numbering system for immunoglobulin constant regions), also contains the conserved N-glycosylation site at asparagine-297, which plays important roles in Fc effector functions and structural stability. This YTE modification strengthens IgG1 binding to the neonatal Fc receptor (FcRn) at the acidic pH (~6.0) of the lysosome, reduces antibody degradation, and promotes recycling to the cell surface; this ultimately extends the serum half-life of the antibody, approximately 69–71 days in infants, by more than twice that of conventional IgG1 monoclonal antibodies, being considerably longer than palivizumab (Figure 1) [6,7,11,20,21,22,23].

Together, targeting antigenic site Ø and incorporating the YTE Fc modification are the defining molecular features that distinguish Nirsevimab from previous RSV monoclonal antibodies. The combination of enhanced neutralizing potency and a substantially prolonged serum half-life enables protection throughout an entire RSV season following a single intramuscular administration, and this represents the principal clinical advantage of Nirsevimab (Figure 2) [19].

### 3.2. Clinical Development Program

The clinical development of Nirsevimab followed a sequential strategy, progressing from proof-of-concept studies to large multinational phase III trials. The phase IIb randomized, placebo-controlled trial (NCT02878330) provided initial evidence by evaluating a single dose of Nirsevimab in healthy preterm infants born between 29 and <35 weeks’ gestation. This study showed significant protection against medically attended RSV-associated lower respiratory tract infection during the 150-day follow-up period after administration and provided the first clinical validation of single-dose, season-long RSV immunoprophylaxis [21].

These findings were subsequently confirmed in the pivotal phase III MELODY trial (NCT03979313), which expanded the assessment of Nirsevimab to healthy late-preterm and term infants born at ≥35 weeks’ gestation. Conducted in 21 countries, MELODY enrolled 1490 infants who were randomized in a 2:1 ratio to receive either a single intramuscular dose of Nirsevimab or placebo, prior to the onset of the local RSV season. Despite operational challenges related to the COVID-19 pandemic, such as altered RSV circulation patterns and enrollment disruptions, the trial successfully met its primary endpoint and demonstrated significant protection against medically attended RSV-associated lower respiratory tract infection [24]. Through the demonstration of robust efficacy in healthy late-preterm and term infants, the MELODY trial expanded the clinical evidence base for Nirsevimab and reinforced its suitability for universal infant RSV prevention.

The Phase II/III MEDLEY trial (NCT03959488) was designed to assess Nirsevimab in infants at increased risk of severe RSV disease, such as preterm infants and those with congenital heart disease or chronic lung disease of prematurity, groups that had previously relied on monthly palivizumab prophylaxis for RSV prevention. In contrast to the preceding trials, the MEDLEY study was designed with an active comparator (palivizumab), given that placebo administration was ethically inappropriate in infants at increased risk of severe RSV disease. The results showed comparable safety and pharmacokinetic profiles between Nirsevimab and palivizumab, supporting Nirsevimab as an alternative preventive strategy in these vulnerable populations [25].

Overall, the clinical development program for Nirsevimab followed a sequential and well-structured approach, progressively expanding its evaluation from preterm infants to all infants, and subsequently to infants at increased risk of severe RSV disease. Consistent demonstration of efficacy and safety across diverse infant populations provided the foundation for regulatory approval by major international health authorities and its subsequent incorporation into national RSV immunization and prevention programs.

### 3.3. Regulatory Approvals

The favorable outcomes of the clinical development program led to the rapid regulatory authorization of Nirsevimab, a major milestone in preventing RSV disease in infants.

Nirsevimab reached an important milestone in RSV prevention with its first regulatory approvals in late 2022. The European Commission granted marketing authorization in October 2022, followed by approval by the UK Medicines and Healthcare Products Regulatory Agency (MHRA) in November 2022 [26,27]. Regulatory approval subsequently expanded to other regions, including the United States, where the U.S. Food and Drug Administration (FDA) approved Nirsevimab in July 2023, followed by recommendations from the Advisory Committee on Immunization Practices (ACIP) and the American Academy of Pediatrics (AAP) supporting its routine clinical use. These regulatory decisions paved the way for incorporating Nirsevimab into national immunization strategies and international clinical recommendations, enabling its implementation as a population-based approach to RSV prevention in infancy [28].

These approvals facilitated the integration of Nirsevimab into routine RSV prevention strategies and supported its adoption across healthcare systems worldwide. The authorization of a single-dose monoclonal antibody represented an important advancement in the practical implementation of RSV immunoprophylaxis [29].

## 4. Clinical Evidence for Nirsevimab Efficacy

Evidence supporting the clinical efficacy of Nirsevimab was established through a comprehensive clinical development program and reinforced by subsequent clinical trials and pooled analyses. Across diverse infant populations, including healthy preterm, term, and high-risk infants, Nirsevimab consistently demonstrated substantial protection against RSV-associated lower respiratory tract infection (LRTI), hospitalization, and severe disease during the first RSV season. Collectively, these findings support its role as an effective immunoprophylactic intervention for routine infant RSV prevention.

The Phase IIb trial (NCT02878330) provided the first proof of concept for the single-dose seasonal immunoprophylaxis. Conducted in healthy preterm infants (29–34 + 6 weeks’ gestation), the study demonstrated a 70.1% reduction in medically attended RSV-associated LRTI (2.6% vs. 9.5%; 95% CI, 52.3–81.2; *p* < 0.001) and a 78.4% reduction in RSV-related hospitalizations (0.8% vs. 4.1%; 95% CI, 51.9–90.3; *p* < 0.001) compared with the placebo. Protection was maintained throughout the 150-day follow-up period, confirming that a single administration could provide coverage for an entire RSV season. Moreover, efficacy remained consistent across geographic regions and against both RSV-A and RSV-B subtypes, supporting the robustness of the observed protective effect. Nevertheless, because the study was limited to healthy preterm infants and follow-up was restricted to one RSV season, its findings could not be directly generalized to the broader infant population or to longer-term protection [21].

Building on these results, the pivotal phase III MELODY trial (NCT03979313) extended the evaluation of Nirsevimab to healthy late-preterm and term infants (≥35 weeks’ gestation). Nirsevimab reduced medically attended RSV-associated LRTI by 74.5% (95% CI, 49.6–87.1; *p* < 0.001), thereby confirming the efficacy observed in the phase IIb study. Although RSV-related hospitalizations occurred less frequently among infants receiving Nirsevimab, the study was not powered to detect statistically significant differences for this secondary endpoint because of the relatively small number of hospitalization events. Protection persisted throughout the 150-day observation period, and further supported the ability of a single dose to provide season-long protection. However, as in the phase IIb trial, efficacy was evaluated during only one RSV season, and the duration of protection beyond this period remained uncertain [24].

Evaluation was subsequently expanded to infants at increased risk of severe RSV disease in the phase II/III MEDLEY trial (NCT03959488), which included infants with congenital heart disease, chronic lung disease of prematurity, and extreme prematurity. Because placebo administration would have been ethically inappropriate in these vulnerable populations, palivizumab served as the active comparator. The trial demonstrated comparable safety and pharmacokinetic profiles between the two monoclonal antibodies, while anti-drug antibodies were detected less frequently in infants receiving Nirsevimab than in those receiving palivizumab (0.4% vs. 3.6%).

It should be noted that the MEDLEY trial was primarily designed to characterize the safety and pharmacokinetic profile of Nirsevimab rather than to establish direct comparative efficacy against palivizumab. Accordingly, the anticipated clinical benefit in high-risk infant populations was inferred largely through pharmacokinetic extrapolation based on exposure–response relationships established in efficacy trials [30].

The effectiveness of Nirsevimab under routine clinical conditions was subsequently evaluated in the large phase IIIb HARMONIE trial (NCT05437510). Conducted in three European countries using a pragmatic design, the study demonstrated an 83.2% reduction in RSV-associated hospitalizations (95% CI, 67.8–92.0; *p* < 0.001) and a 75.7% reduction in very severe RSV-associated LRTI (95% CI, 32.8–92.9; *p* = 0.004). Despite the pragmatic design’s advantage in enhancing external validity, this approach may also lead to greater clinical heterogeneity than in controlled double-blind randomized trials. Moreover, because the study was conducted only in three European countries and captured outcomes over one RSV season, further studies are required to determine the consistency and durability of these findings across different epidemiological settings and over subsequent seasons [31].

Integrated analyses of randomized clinical trials strengthened this evidence. A pooled analysis of the phase IIb and MELODY studies demonstrated relative risk reductions of 79.5% for medically attended RSV-associated LRTI, 77.3% for RSV-related hospitalization, and 86.0% for very severe RSV disease. Beyond reducing RSV-specific outcomes, Nirsevimab also decreased all-cause respiratory hospitalizations, medically attended lower respiratory tract infections, outpatient visits, and antibiotic prescriptions, suggesting broader clinical and healthcare benefits [22]. Beyond this, a post hoc analysis of the MELODY trial demonstrated that Nirsevimab selectively reduced RSV-associated LRTIs, including RSV coinfections with rhinovirus/enterovirus, without affecting the incidence of infections caused by other respiratory viruses. These findings provide reassuring evidence that RSV immunoprophylaxis does not promote viral replacement and further support Nirsevimab’s targeted mechanism of action [32].

The systematic review and meta-analysis by Lien et al. provided additional support [33]. This confirmed substantial protection against severe RSV disease and maintenance of an excellent safety profile. Nirsevimab significantly reduced the risk of RSV-associated hospitalization (pooled OR 0.19; 95% CI, 0.13–0.30) and severe RSV disease requiring intensive care or respiratory support (pooled OR 0.23; 95% CI, 0.12–0.44). Importantly, the consistency of effects across studies, reflected by the absence of significant between-study heterogeneity (I^2^ = 0%), further strengthens the consistency and external validity of these findings [33].

Overall, the accumulated clinical evidence establishes Nirsevimab as a highly effective intervention for preventing severe RSV disease during infancy. The consistency of efficacy across randomized trials, high-risk populations, and pragmatic clinical studies provides a robust evidence base supporting its widespread implementation (Table 2).

Real-world evidence generated following the introduction of Nirsevimab into routine clinical practice has consistently confirmed the high effectiveness observed in randomized clinical trials. In diverse healthcare systems in North America, South America, and Europe, Nirsevimab demonstrated substantial protection against RSV-associated hospitalization, severe lower respiratory tract infection (LRTI), and intensive care unit (ICU) admission. Although effectiveness estimates vary according to study design, outcome definitions, RSV epidemiology, and immunoprophylaxis coverage, the overall consistency of findings strongly supports the external validity and generalizability of the clinical trial program.

In the United States, a multicenter test-negative study across six healthcare systems during the 2023–2024 RSV season reported 77% effectiveness against RSV-associated emergency department visits and 98% against RSV-related hospitalizations [35]. Similarly, in Alaska native children, Nirsevimab reduced medically attended RSV illness by 76% and RSV-associated hospitalization by 89% during the first RSV season, while effectiveness against medically attended RSV illness reached 88% in high-risk children entering their second RSV season. These findings demonstrate that the substantial protection observed in clinical trials is maintained in routine clinical practice, including populations at increased risk of severe disease [36].

Comparable effectiveness has been reported internationally. Chile was the first country in the Southern Hemisphere to implement universal Nirsevimab immunoprophylaxis, nationwide, effectiveness reached 76.41% against RSV-related hospitalization and 84.94% against ICU admission and prevented an estimated of 30 RSV-related hospitalizations per 1000 infants. The Chilean experience is particularly important because it represents the first nationwide universal implementation of Nirsevimab out of Europe and North America, demonstrating that the benefits observed in clinical trials can be reproduced at a national public health level [37]. In Europe, a population-based cohort study from Madrid demonstrated sustained protection against RSV-related hospitalization with effectiveness of 93.6% at 30 days and 87.6% at 150 days, while protection against ICU admission exceeded 90%, and provided further evidence of prolonged clinical effectiveness throughout a single RSV season.

Consistent results were also reported in France and Italy, where effectiveness against RSV-associated hospitalization was estimated to be 78.3% and 84.4%, respectively, reinforcing the reproducibility of these findings across different healthcare settings [38,39].

Although real-world evidence provided important insights into the effectiveness of Nirsevimab in routine clinical settings, the predominantly observational nature of these studies introduces potential limitations, including residual confounding, selection bias, and variability in healthcare-seeking behavior and healthcare utilization. In addition, differences in RSV epidemiology, including seasonal patterns and viral circulation, variations in immunization and immunoprophylaxis coverage, and heterogeneity in outcome definitions limit the comparability of results across countries and healthcare systems. Despite these limitations, the consistency of findings in multiple independent real-world studies provides strong support for the effectiveness of Nirsevimab in routine clinical practice (Table 3).

The accumulated real-world evidence has recently been strengthened by a systematic review and meta-analysis encompassing 32 observational studies [41]. Compared with infants who did not receive immunoprophylaxis, Nirsevimab administration was associated with significant reductions in RSV-related hospitalization (OR 0.17; 95% CI, 0.12–0.23), intensive care unit admission (OR 0.19; 95% CI, 0.12–0.29), and RSV-associated lower respiratory tract infection (OR 0.25; 95% CI, 0.19–0.33) during the first year of life. However, no significant difference was observed in the length of hospital stay among infants requiring inpatient care. Overall, this meta-analysis confirms that the high effectiveness of Nirsevimab demonstrated in randomized clinical trials translates into routine clinical practice, despite heterogeneity in patient populations and healthcare systems [41].

## 5. Safety Profile of Nirsevimab

Nirsevimab demonstrated a highly favorable safety profile in randomized clinical trials, pooled analyses, and real-world pharmacovigilance studies. In studies, adverse events occurred at similar frequencies in the Nirsevimab and control groups, and were predominantly mild to moderate in severity, and were rarely considered treatment-related. Importantly, no consistent safety signals, including anaphylaxis, severe hypersensitivity reactions, vaccine-associated enhanced respiratory disease (VAERD), or other unexpected adverse events, have emerged during either clinical development or routine clinical use.

The Phase IIb trial provided the first clinical evidence that supports the safety of Nirsevimab and demonstrates adverse event rates comparable to those observed with placebo, fewer serious adverse events in treated infants, and no treatment-related serious adverse events or hypersensitivity reactions [21]. These findings were subsequently reinforced by the pivotal Phase III MELODY trial, in which the incidence of adverse events and serious adverse events was comparable between treatment groups, with grade ≥3 adverse events remaining uncommon (3.6% with Nirsevimab vs. 4.3% with placebo). Treatment-emergent adverse events of special interest were rare, and no cases of anaphylaxis were reported, demonstrating that extension of antibody half-life through Fc engineering did not compromise safety in healthy infants [24]. Pooled analyses of clinical trials further confirmed the absence of significant differences in mortality, adverse events of special interest, or serious adverse events compared with control groups, including palivizumab [42].

Likewise, a recent systematic review and meta-analysis of six randomized controlled trials reported no significant increase in treatment-emergent adverse events in infants receiving Nirsevimab (pooled OR 0.91; 95% CI, 0.61–1.34). Although moderate between-study heterogeneity was observed (I^2^ = 61.9%), likely reflecting differences in study populations and adverse event reporting, the findings consistently demonstrated a safety profile comparable to that of the control groups [33].

Long-term follow-up provided additional reassurance regarding the durability of the safety profile. Treatment-related adverse events remained infrequent and were largely limited to mild injection-site reactions or transient rash, with no relationship identified between Nirsevimab exposure and serious adverse events. Moreover, no delayed safety signals were detected during extended follow-up [43].

Evidence from post-marketing surveillance closely mirrored the findings of clinical trials. During Spain’s 2023–2024 national Nirsevimab immunization campaign, adverse events were reported at a rate of only 23.1 cases per 100,000 administered doses, while no new safety concerns, including immune complex disease and antibody-dependent enhancement, were identified [44]. Concerns regarding vaccine-associated enhanced respiratory disease were also addressed in follow-up analyses, which showed no increase in RSV disease severity, hospitalization, or medically attended illness in infants previously exposed to Nirsevimab compared with placebo [45].

The absence of vaccine-associated enhanced respiratory disease is particularly reassuring given the historical experience with the formalin-inactivated RSV vaccine in the 1960s, which raised longstanding concerns regarding immune-mediated disease enhancement following RSV immunization.

## 6. Efficacy and Safety in Immunocompromised Children

Evidence regarding Nirsevimab in immunocompromised children remains limited but is encouraging. The open-label phase II MUSIC trial evaluated Nirsevimab in 100 immunocompromised children aged ≤24 months, including primary immunodeficiency, solid organ or hematopoietic stem cell transplantation, HIV infection, and under immunosuppressive therapy [46]. A single weight-based dose achieved serum concentrations at day-151 comparable to those previously associated with protection in healthy infants, and no protocol-defined medically attended RSV-associated lower respiratory tract infections were observed during follow-up.

Nirsevimab was generally well tolerated, and only eight treatment-related adverse events were reported in six children, none of which were considered serious. During the follow-up period, three deaths occurred. However, all were assessed as unrelated to Nirsevimab administration. Although anti-drug antibodies were detected in 11 participants, their impact on pharmacokinetics was limited, and they did not appear to influence the overall safety profile of the drug. Furthermore, increased clearance of Nirsevimab was observed in a subgroup of 14 children, primarily in patients with conditions associated with protein loss [46]. To date, the clinical relevance of this observation has not been established.

Although the study was not powered to evaluate clinical efficacy and did not include a comparator group, the pharmacokinetic data and the highly favorable safety profile provide strong scientific support for the use of Nirsevimab in this particularly vulnerable patient population. Nevertheless, larger prospective and controlled studies are required to further establish its clinical effectiveness, duration of protection, and precise role in immunocompromised children [43].

The safety profile was similarly reassuring. Although adverse events were common (81%), reflecting the underlying medical complexity of the study population, treatment-related events were infrequent (6%), generally mild, although grade ≥3 events occurred in 35% of participants, most occurred more than 30 days after administration and were considered unlikely to be related to Nirsevimab. Early post-dose reactions were rare, hypersensitivity events were limited to mild cutaneous manifestations, and no treatment-related deaths or RSV-associated deaths were reported [46].

As Nirsevimab use expands, further data are needed to evaluate safety in underrepresented populations, repeated administration across consecutive RSV seasons, potential interactions with routine childhood vaccination schedules, and the combined use of maternal RSV vaccination and infant immunoprophylaxis.

## 7. Clinical Implementation of Nirsevimab

The transition of Nirsevimab from clinical trials to routine clinical practice required clear recommendations on the populations most likely to benefit, the optimal timing of administration, and integration into existing immunization programs. Accordingly, international scientific societies and regulatory agencies issued detailed recommendations, which are updated as new evidence emerges from real-world clinical implementation. Current recommendations support the administration of Nirsevimab to all infants entering their first RSV season who have not received adequate protection via maternal RSV vaccination. For infants born during the RSV season, Nirsevimab should ideally be administered within the first week of life, ideally prior to discharge from the maternity unit, to ensure protection during the period of highest vulnerability [47].

Eligibility decisions increasingly incorporate infant age and risk profile and maternal RSV vaccination status, reflecting the complementary roles of maternal immunization and monoclonal antibody prophylaxis. Effective implementation therefore requires coordination between obstetric services, maternity units, pediatric teams, and public health programs to ensure appropriate identification of eligible infants and timely delivery of preventive interventions. In practice, such a coordination mechanism could be established through an integrated care pathway initiated during antenatal care, with systematic documentation of maternal RSV vaccination status and subsequent communication of this information to the maternity and neonatal teams at delivery. At birth, maternal vaccination status, the interval between maternal vaccination and delivery, gestational age, and infant risk factors can be used to determine eligibility for Nirsevimab. Among eligible infants born during the RSV season, administration before hospital discharge is an important opportunity for timely immunoprophylaxis. For those who do not receive Nirsevimab before discharge or who become eligible later in the RSV season, appropriate linkage to pediatric primary care and immunization services is essential to ensure timely administration. Shared electronic health records or standardized documentation of maternal RSV vaccination and infant Nirsevimab administration could help reduce both missed opportunities for immunoprophylaxis and unnecessary dual administration. At the public health level, centralized monitoring of RSV epidemiology, product availability, and immunization coverage could support procurement planning and the appropriate timing of program implementation.

Although a single dose provides protection throughout one RSV season for most infants, a second seasonal dose of 200 mg is recommended for selected children at increased risk of severe RSV disease entering their second RSV season. These are children with chronic lung disease of prematurity requiring ongoing medical therapy, severe immunocompromising conditions, cystic fibrosis with severe lung disease or poor nutritional status, and certain Indigenous populations in accordance with national recommendations [47].

### 7.1. Dosage and Administration

The prolonged serum half-life of Nirsevimab allows a simplified weight-based dosing regimen that provides protection throughout a RSV season. Infants weighing <5 kg receive 50 mg, whereas those weighing ≥5 kg receive 100 mg, administered as a single intramuscular injection before or during their first RSV season. For children aged 8–19 months who remain at increased risk of severe RSV disease and enter their second RSV season, a single 200 mg dose, administered as two 100 mg intramuscular injections, is recommended [47].

Nirsevimab is administered as a single intramuscular (IM) injection, typically delivered into the anterolateral region of the thigh in infants prior to the onset of the respiratory syncytial virus (RSV) season [21]. Its simple weight-based dosing regimen is a key practical advantage that facilitates integration into routine clinical practice and improves the feasibility of large-scale RSV immunoprophylaxis, while minimizing the risk of dosing and administration errors.

### 7.2. Timing According to RSV Seasonality

The effectiveness of Nirsevimab depends not only on appropriate patient selection but also on administration at the optimal time relative to local RSV circulation. Aligning prophylaxis with the onset of the RSV season ensures that protective antibody concentrations are maintained throughout the period of greatest viral activity, thereby maximizing protection against severe RSV disease, while avoiding unnecessary administration out of the epidemic period. In temperate regions of the Northern Hemisphere, Nirsevimab is generally administered shortly before the RSV season, typically between October and March, whereas in the Southern Hemisphere its administration is scheduled according to the corresponding regional seasonal pattern [47]. However, RSV epidemiology varies considerably across geographic settings. Temperate regions usually exhibit predictable seasonal epidemics, but tropical and subtropical regions often experience prolonged, irregular, or year-round viral circulation [47] Indeed, a global analysis of RSV seasonality showed that RSV season duration was, on average, 1.06 months longer in subtropical and 1.65 months longer in tropical regions than in temperate regions [48]. These findings highlight the importance of incorporating local climatic and epidemiological patterns when determining the optimal timing of Nirsevimab administration, particularly in regions where RSV circulation does not follow a clearly defined seasonal pattern.

The COVID-19 pandemic further altered RSV epidemiology and disrupted the traditional seasonal pattern observed in many countries. Widespread implementation of non-pharmaceutical interventions (NPIs) substantially reduced RSV transmission, leading to an accumulation of immunologically naïve infants and young children. After relaxing these measures, many countries reported delayed, off-season, or unusually intense RSV outbreaks [34]. These epidemiological changes underscore the importance of continuous RSV surveillance to guide the timing of Nirsevimab administration and to ensure that immunoprophylaxis remains aligned with evolving patterns of viral circulation [34,47]. These observations highlight that successful implementation of Nirsevimab requires a flexible, region-specific approach, with administration timing guided by local RSV surveillance rather than fixed calendar schedules alone.

## 8. Potential Impact of Nirsevimab on Post-RSV Sequelae

Respiratory syncytial virus (RSV) infection during early life has long been associated with an increased risk of recurrent wheezing and childhood asthma. Proposed mechanisms include airway epithelial injury, persistent airway inflammation, enhanced bronchial hyperresponsiveness, and dysregulated immune responses following severe infection. Despite the well-established association between severe RSV infection during infancy and the subsequent development of recurrent wheezing or asthma, the causal relationship remains the subject of ongoing scientific debate. It remains unclear whether RSV directly contributes to the pathogenesis of long-term respiratory disorders or whether it primarily identifies children with pre-existing genetic or immunological susceptibility to their development [35,49].

Although the available evidence suggests a potential association between Nirsevimab-mediated prevention of RSV disease and reduced certain subsequent respiratory outcomes, these findings should be interpreted with caution. The current evidence base comes predominantly from observational analyses and varies across study populations, follow-up periods, and definitions of post-RSV outcomes. Consequently, a causal relationship between Nirsevimab administration and reduced long-term respiratory sequelae cannot yet be established.

Emerging evidence suggests a potential reduction in early-life wheezing following Nirsevimab administration. In a large observational study during the 2023–2024 RSV season, infants who received Nirsevimab had a 27% lower risk of wheezing episodes during 12 months of follow-up compared with untreated infants (HR 0.73; 95% CI, 0.58–0.93). These findings raise the possibility that early RSV prevention may reduce subsequent airway inflammation and respiratory morbidity. However, it remains unclear whether this represents true disease modification or simply a delay in primary RSV infection [49]. Although these findings are promising, they originate from an observational study and should therefore be interpreted cautiously, given the possibility of residual confounding and other sources of bias.

Additional evidence from studies evaluating RSV monoclonal antibody prophylaxis provides further, albeit inconclusive, support for potential long-term respiratory benefits. A meta-analysis of eight studies included 11,195 infants and reported a non-significant reduction in recurrent wheezing or asthma overall (RR 0.60; 95% CI, 0.31–1.16); significant protective effects, however, were observed in sensitivity analyses excluding low-quality studies (RR 0.42; 95% CI 0.22 to 0.80, *p* = 0.008) and in moderately preterm infants (32 to <36 weeks’ gestation; RR 0.35; 95% CI 0.14 to 0.86, *p* = 0.02) [50]. The heterogeneity of the available studies, including differences in study design, population characteristics, follow-up duration, and definitions of respiratory outcomes, may partly explain the conflicting observations reported in studies. Therefore, current evidence does not yet allow definitive conclusions about whether RSV immunoprophylaxis can alter the natural history of chronic respiratory diseases.

Long-term prospective studies are needed to determine whether Nirsevimab can alter the natural history of RSV-associated respiratory disease. Future research should include follow-up into school age and incorporate clinical outcomes, pulmonary function testing, and quality-of-life measures to define the potential role of RSV immunoprophylaxis in preventing chronic respiratory morbidity. Particular emphasis should be placed on using predefined respiratory outcomes and appropriate comparator groups to determine whether the observed associations reflect true disease modification or are influenced by confounding factors, including underlying host susceptibility and the timing or severity of RSV infection.

## 9. Nirsevimab Resistance and Viral Escape

The potential emergence of RSV resistance represents an important consideration as Nirsevimab is implemented on a population scale. Unlike vaccines, monoclonal antibodies exert a highly specific selective pressure by targeting a defined antigenic epitope of the RSV F protein. Consequently, continued genomic surveillance is essential to identify escape mutations that could compromise Nirsevimab’s long-term effectiveness [6].

RSV undergoes continuous genetic evolution, with its two major antigenic subtypes, RSV-A and RSV-B, co-circulating and showing variable predominance across seasons and regions [51]. Despite ongoing RSV evolution, the fusion (F) protein shows relatively low genetic diversity, while the Nirsevimab binding site within antigenic site Ø of the prefusion F protein remains highly conserved.

In a prospective molecular surveillance analysis of 5675 RSV F-protein sequences collected between 2015 and 2021, all 25 amino acid positions comprising the Nirsevimab binding site in RSV-A and 22 of the 25 positions in RSV-B were highly conserved. The greater sequence variability observed in RSV-B was primarily associated with the emergence and predominance of the I206M: Q209R polymorphism. However, Nirsevimab retained its neutralizing activity against this prevalent variant, while variants with reduced susceptibility to Nirsevimab-mediated neutralization remained rare (<1%) and did not increase in frequency during the 2015–2021 period [52].

Data from pivotal clinical trials are reassuring. Molecular and phenotypic analyses of RSV isolates obtained during the Phase IIb and MELODY trials demonstrated that more than 99% of circulating viruses remained susceptible to Nirsevimab [24,53]. In RSV-A, no major substitutions within the Nirsevimab binding site were observed among cases of medically attended MA RSV LRTI or RSV-related hospitalization, while the K209R substitution, detected in two participants with exploratory RSV endpoints, did not affect susceptibility to Nirsevimab. Although greater genetic variability within the Nirsevimab binding site was observed in RSV-B, the most frequently detected substitutions I206M: Q209 and Q209R, as well as S211N, remained effectively neutralized by Nirsevimab. Notably, RSV-B variants carrying the Q209R and I206M: Q209R substitutions exhibited increased susceptibility to Nirsevimab. Molecular modeling suggested that the Q209R substitution may enhance electrostatic interactions with Nirsevimab, thereby stabilizing the F protein in its prefusion conformation and, consequently, increasing neutralization potency. In contrast, only three substitutions, I64T, K68E, and N208S, detected in RSV-B isolates from two Nirsevimab recipients in the Phase IIb trial, were associated with phenotypic resistance and >200-fold increase in IC50. These variants emerged in participants who had received a dose subsequently considered suboptimal, were not detected in subsequent clinical trials or molecular surveillance studies, and no resistance-associated substitutions were identified beyond 150 days after Nirsevimab administration. Overall, these findings indicate that amino acid substitutions within the Nirsevimab-binding site do not necessarily confer reduced susceptibility to neutralization and underscore the need for integrated genotypic and phenotypic characterization to assess the clinical and virological significance of potential escape variants [53,54].

However, real-world surveillance identified sporadic emergence of resistance-associated substitutions (RASs), particularly in RSV-B breakthrough infections following Nirsevimab administration [46].

The multicenter POLYRES-2 study conducted in France during the 2024–2025 RSV season provided important insights into the evolution of escape variants in routine clinical practice. In 1023 infants with RT-PCR-confirmed RSV infection, complete genomic data were available for 858 cases, including 419 infections occurring after Nirsevimab exposure and 439 in Nirsevimab-naïve infants. RASs were detected exclusively in breakthrough infections, with a higher frequency in RSV-B compared with RSV-A (12.5% vs. 1.0%) [55]. Despite detecting escape mutations, most breakthrough infections after Nirsevimab administration were still caused by RSV strains lacking resistance-associated substitutions. This suggests that additional factors, including exposure to high viral loads, host-related characteristics, or declining antibody concentrations toward the end of the protection period, may also contribute to breakthrough infections. Escape-associated substitutions were primarily located within or near antigenic site Ø of the RSV F protein, particularly around residue 208 (F: N208D, F: N208I, F: N208K, F: N208S, or F: N208Y), with additional changes involving residues implicated in antibody binding. Importantly, no resistance-associated substitutions were identified in Nirsevimab-naïve infants.

Current evidence therefore indicates that resistance remains an emerging virological phenomenon rather than a clinically significant limitation of Nirsevimab. Although escape variants can occur, their overall frequency remains low; they have predominantly been identified in breakthrough infections, and there is currently no evidence of widespread transmission or reduced population-level protection. The absence of resistance-associated variants in infants protected through maternal RSV vaccination also raises the possibility that polyclonal antibody responses generated by maternal immunization may exert different selective pressures compared with a monoclonal antibody targeting a single conserved epitope; however, this hypothesis requires further investigation. Continued genomic surveillance, combined with phenotypic susceptibility testing and clinical monitoring, will be essential to detect changes in RSV evolution and evaluate their potential impact on the long-term effectiveness of Nirsevimab programs. Importantly, identifying an amino acid substitution within the Nirsevimab binding site alone does not establish clinically meaningful resistance. Determining its functional and clinical relevance requires phenotypic confirmation, along with assessment of its association with breakthrough-infection characteristics [53]. Particular attention should be directed toward RSV-B strains, given the higher frequency of escape-associated substitutions observed in recent surveillance studies, although the biological mechanisms underlying this apparent subtype-specific susceptibility remain incompletely understood. To date, the emergence of resistance-associated variants has not translated into a measurable reduction in the overall clinical effectiveness of Nirsevimab against severe RSV disease, but ongoing monitoring will be critical to ensure the durability of this prevention strategy as global implementation expands [55].

## 10. Nirsevimab and Maternal RSV Vaccination: Complementary Approaches to Infant RSV Prevention

Maternal RSV vaccination and Nirsevimab are complementary strategies that substantially reduce RSV-associated morbidity, but differ in their mechanisms, timing of protection, and dependence on maternal and placental factors. Maternal RSV vaccination induces active maternal immunity, generating neutralizing antibodies against the prefusion RSV F protein that are transferred to the fetus through neonatal Fc receptor-mediated placental transport. Therefore, maternal vaccination depends on adequate vaccine uptake, timing during pregnancy, and efficient transplacental antibody transfer, whereas Nirsevimab provides predictable protection independent of maternal immune status, placental function, or gestational age [56].

The efficacy of maternal RSV vaccination was established in the phase III MATISSE trial, which demonstrated 81.8% (99.5% CI, 40.6–96.3) efficacy against severe medically attended RSV-associated lower respiratory tract infection (LRTI) within 90 days after birth and 69.4% (97.58% CI, 44.3–84.1) through 180 days [56]. Real-world evidence subsequently supported these findings. In Argentina, the BERNI study reported effectiveness of 78.6% (95% CI, 62.1–87.9) against severe RSV disease during the first three months of life, with protection remaining substantial through six months, while effectiveness against severe RSV-associated hospitalization reached 76.9% (95% CI, 45.0–90.3) [38]. Similarly, a multicenter study from the United Kingdom demonstrated a 61% reduction in RSV-associated infant hospitalizations up to six months of age (95% CI, 38–75), with even greater effectiveness in infants younger than three months (76%; 95% CI, 54–87) [57]. Regarding safety, the MATISSE trial did not identify any major safety concerns for either mothers or infants, and the overall incidence of adverse events was comparable between the vaccine and placebo groups. However, given that vaccination is administered during pregnancy, continued evaluation of pregnancy-related outcomes, including preterm birth, remains particularly important and warrants ongoing post-licensure pharmacovigilance and real-world safety surveillance [56,58].

An important limitation of maternal immunization is its reliance on efficient transplacental antibody transfer, which occurs predominantly during the third trimester. Consequently, the interval between vaccination and delivery, as well as gestational age at birth, influences infant protection. Recent comparative data from France showed that maternal vaccination provided protection comparable to Nirsevimab when administered at least eight weeks before delivery, whereas shorter vaccination-to-delivery intervals were associated with higher RSV hospitalization rates. This limitation is particularly relevant for preterm infants, who receive reduced quantities of maternally derived IgG, because much of placental antibody transfer occurs late in pregnancy. Nirsevimab bypasses this limitation by providing direct infant protection and is therefore particularly advantageous when maternal vaccination is not performed, is administered shortly before delivery, or when placental antibody transfer is likely to be insufficient. Nevertheless, these findings require cautious interpretation, given their derivation from an observational study and the potential influence of residual confounding and other unmeasured sources of bias. Consequently, current evidence remains insufficient to establish the superiority of one preventive strategy over the other [57].

In a comparative cohort study of 42,560 infants, passive immunization with Nirsevimab was associated with a lower risk of RSV-associated lower respiratory tract infection (LRTI) hospitalization compared with maternal RSVpreF vaccination. During follow-up, RSV-related hospitalization occurred less frequently in infants receiving Nirsevimab (44.1%) than in those protected through maternal vaccination (55.9%), corresponding to a 26% relative reduction in risk (adjusted HR 0.74; 95% CI, 0.61–0.88). Nirsevimab was also associated with reduced rates of severe outcomes, including pediatric intensive care unit admission, mechanical ventilation, and oxygen requirement [59]. More recent data also provide the first prospective randomized evidence regarding the sequential administration of the two strategies. In an interim analysis of a phase 4 randomized trial, maternal RSVpreF vaccination and infant Nirsevimab administration, given either individually or sequentially, demonstrated favorable safety profiles, with no treatment-related serious adverse events reported in mothers or infants [60]. Maternal RSVpreF vaccination resulted in a substantial increase in maternal RSV-A and RSV-B neutralizing antibody transfer. High RSV neutralizing antibody titers were also observed in infants following maternal vaccination, Nirsevimab administration, or sequential exposure to both interventions. However, these findings represent interim immunogenicity and safety data with limited follow-up and do not yet establish an additional clinical benefit from the combined or sequential use of the two strategies [60].

Despite the potentially complementary nature of the two approaches, maternal RSV vaccination and infant Nirsevimab are generally not required concurrently for most infants. Administration of Nirsevimab following maternal vaccination may be considered in selected circumstances, such as when delivery occurs <14 days after maternal vaccination, when an inadequate maternal immune response or transplacental antibody transfer is anticipated, when accelerated waning of maternal antibodies is expected, or when the infant is at particularly high risk of severe RSV disease [61].

Overall, the available evidence supports both maternal RSV vaccination and Nirsevimab administration as highly effective interventions for the prevention of severe RSV disease during the first months of life. In settings where reliable postnatal follow-up and timely access to infant immunoprophylaxis cannot be ensured, or where the substantially higher per-dose cost of Nirsevimab limits affordability, maternal RSV vaccination may represent a more feasible preventive strategy, as the intervention is completed during antenatal care and protection is conferred to the infant from birth. This consideration may be particularly relevant in resource-limited settings and low- and middle-income countries (LMICs), where the higher acquisition cost of Nirsevimab, procurement and distribution requirements, and limited access to postnatal healthcare services may challenge the universal implementation of infant immunoprophylaxis. In such healthcare systems, maternal vaccination may therefore be more operationally feasible, provided adequate antenatal care coverage is available and vaccination can be administered within the recommended gestational window and sufficiently before delivery. The optimal integration of these two complementary strategies into national prevention programs will likely be determined by future comparative studies, accumulating real-world evidence, and the specific characteristics and priorities of each healthcare system.

## 11. Implementation Challenges

Despite its considerable clinical advantages, several practical challenges remain. Implementing the program requires reliable procurement systems, adequate funding, and healthcare professional education. Accurate documentation of maternal vaccination status may not always be readily available, particularly when prenatal care is fragmented, increasing the risk of inappropriate administration or missed prophylaxis. Furthermore, ensuring equitable access for infants born in rural or resource-limited settings represents an ongoing challenge. In low- and middle-income countries, financial constraints, competing public health priorities, and limited healthcare infrastructure may delay widespread implementation despite the substantial burden of RSV disease. Addressing these logistical and organizational barriers will be essential to maximize population coverage and ensure that the clinical benefits demonstrated in trials and real-world studies translate into meaningful reductions in RSV-related morbidity and healthcare utilization. Furthermore, the implementation of Nirsevimab requires effective coordination among maternity units, neonatal departments, pediatric services, and national immunization programs to ensure timely administration prior to the newborn’s discharge from the maternity hospital.

### 11.1. Cost-Effectiveness and Public Health

Economic evaluations consistently suggest that Nirsevimab can substantially reduce the clinical and economic burden of RSV, although its cost-effectiveness depends on product price, local RSV epidemiology, healthcare costs, and implementation strategy. A US modeling study estimated that immunizing 50% of the annual birth cohort would prevent 107,253 outpatient visits, 38,204 emergency department visits, and 14,341 RSV-related hospitalizations annually, with an incremental cost-effectiveness ratio (ICER) of US$153,517 per quality-adjusted life year (QALY) gained [62]. Similarly, a Japanese decision-analytic model reported that universal infant immunization with Nirsevimab could reduce approximately 50% of RSV-associated healthcare events and was cost-effective from both healthcare payer and societal perspectives, particularly when indirect costs were included [63].

Canadian studies identified Nirsevimab for high- and moderate-risk infants and seasonal programs with catch-up vaccination as the most cost-effective strategies at current prices, whereas universal infant immunization was generally not cost-effective unless acquisition costs were substantially reduced [64].

Moreover, a cost-effectiveness analysis from Hong Kong used a Markov model to evaluate year-round, seasonal, and catch-up Nirsevimab immunization strategies in newborns. All Nirsevimab strategies reduced RSV-related lower respiratory tract infections and QALY losses, with the catch-up strategy providing the greatest health benefit [65].

Overall, current evidence supports Nirsevimab as an effective and potentially cost-effective strategy for reducing RSV-related morbidity and healthcare burden. However, its cost-effectiveness is not a fixed parameter but depends on multiple factors, including product price, local RSV epidemiology, hospitalization costs, and the implementation strategy adopted (universal versus targeted immunoprophylaxis). Consequently, the long-term public health impact of Nirsevimab will depend on sustainable financing, equitable access, appropriate integration with maternal RSV vaccination programs, and the adaptation of prevention strategies to the specific characteristics of each healthcare system and local epidemiological context.

### 11.2. Global Access and Implementation Challenges

The global impact of RSV prevention with Nirsevimab will depend not only on its clinical effectiveness but also on the ability of health systems to ensure timely, affordable, and equitable delivery, particularly in low- and middle-income countries (LMICs). These settings carry a disproportionate burden of RSV-related morbidity and mortality, yet face significant challenges related to healthcare access, infrastructure, and available resources. RSV-associated in-hospital case fatality rates are substantially higher in LMICs compared with that of high-income countries, reflecting limited access to supportive care, delayed presentation to healthcare facilities, and constrained medical resources. Moreover, hospital-based data underestimates the true burden of RSV mortality, as only a minority of RSV-attributable deaths occur in healthcare facilities, with the majority occurring in the community, particularly in low-income countries [66].

Supporting these modelling findings, real-world evidence from the Spanish NIRSE-GAL study showed that nationwide implementation of Nirsevimab was associated with an 85.9% reduction in RSV-related hospitalizations during the first RSV season, and significant decrease in non-RSV respiratory hospitalizations and outpatient visits. These findings indicate that, when supported by adequate organization, financing, and high programme uptake, universal Nirsevimab immunoprophylaxis can be successfully implemented at the population level while translating clinical trial efficacy into substantial public health benefits [44].

Given that infants younger than six months experience the highest risk of severe RSV disease and death, they represent the key population for preventive interventions. Early protection is particularly important, as most RSV-related hospitalizations in early infancy occur within the first three months of life. Modeling studies suggest that effective passive immunization strategies could prevent hundreds of thousands of RSV-related hospitalizations and tens of thousands of deaths globally each year. However, achieving these benefits will require overcoming substantial implementation barriers [66].

Evidence from LMICs indicates that both maternal RSV vaccination and infant immunization with long-acting monoclonal antibodies (mAbs) are considered feasible and acceptable when integrated into existing healthcare platforms. Nevertheless, concerns remain regarding vaccine hesitancy, misinformation, access to antenatal care, and the challenges of delivering maternal vaccination within a specific gestational window. For infant mAbs key barriers include additional demands on existing immunization schedules, misconceptions regarding their use only in high-risk infants, and, most importantly, product affordability and availability. Stakeholders generally favored year-round immunization approaches, as these may reduce missed opportunities and simplify program delivery compared with seasonal strategies.

Ultimately, no single RSV prevention strategy will be appropriate for all settings. Successful implementation will require adaptation to local epidemiology, healthcare capacity, and community needs. In LMICs, the choice between maternal RSV vaccination, infant Nirsevimab, or a combined or targeted approach should be tailored to the capacity of the local health system to deliver the intervention with high and timely coverage. In settings with high antenatal care coverage and adequate access to vaccination services for pregnant women, maternal RSV vaccination can leverage existing maternal immunization platforms.

Conversely, where antenatal care coverage is limited or access during the appropriate gestational window is inadequate, delivery of Nirsevimab through maternity, neonatal, or infant immunization services may provide an alternative delivery strategy, provided that adequate supply and sustainable financing can be ensure. In resource-constrained settings, targeted approaches may also be considered for infants at increased risk of severe RSV disease or during periods of high RSV circulation. Accordingly, policy decisions should consider not only comparative clinical effectiveness but also affordability, supply security, antenatal and birth-facility coverage, cold-chain capacity and programmatic delivery requirements, local RSV seasonality, and available cost-effectiveness and disease-burden data. Strengthening immunization systems, improving access to antenatal and infant health services, ensuring sustainable financing, addressing vaccine confidence, and generating local disease burden and cost-effectiveness data will be essential to achieve equitable RSV prevention worldwide [67]. The long-term success of Nirsevimab-based immunoprophylaxis programs will depend on continuous evaluation of their effectiveness in real-world clinical settings, equitable access for all infants, sustainable funding, and regular updates of national recommendations based on emerging epidemiological and health economic evidence.

## 12. Future Perspectives

The widespread introduction of Nirsevimab represents a significant milestone in RSV prevention and has shifted the focus of research from demonstrating short-term clinical efficacy toward evaluating its long-term population-level impact. While clinical trials and real-world studies have consistently demonstrated high effectiveness and a favorable safety profile, continued post-implementation surveillance will be essential to assess the durability of protection across multiple RSV seasons, evaluate effectiveness in specific vulnerable populations, and determine its performance across diverse epidemiological settings. The evaluation of long-term protection and effectiveness in specific populations will represent a key priority for future research.

A major unresolved issue is the potential effect of widespread RSV immunoprophylaxis on viral transmission and epidemiology. By reducing RSV infections during early infancy, large-scale Nirsevimab use may influence transmission patterns, shift the age at primary infection, modify seasonal dynamics, or alter the intensity of future RSV epidemics. Long-term epidemiological surveillance, supported by mathematical modelling, will therefore be required to understand how changes in population immunity may affect RSV circulation and disease burden over time.

The ongoing evolution of RSV also represents an important area for future investigation. The emergence of Nirsevimab-resistant variants highlights the need for integrated genomic and phenotypic surveillance to monitor changes in viral susceptibility and evaluate their potential clinical significance. Although currently identified escape variants have not compromised population-level effectiveness, sustained monoclonal antibody use may exert selective pressure on RSV evolution.

Future research should also focus on optimizing the role of Nirsevimab within comprehensive RSV prevention programs. Comparative evaluations of maternal RSV vaccination and infant monoclonal antibody prophylaxis will be critical to identify the most effective and economically sustainable strategies across different healthcare settings. Particular attention should be given to populations at increased risk of severe RSV disease, including infants with underlying conditions or limited passive maternal antibody protection. In addition, developing next-generation monoclonal antibodies that target alternative RSV epitopes or use combination approaches may enhance protection and increase resilience against future viral evolution.

Ultimately, Nirsevimab’s global impact will depend not only on scientific advances but also on successful implementation as a public health intervention. Affordability, equitable access, healthcare system capacity, and sustainable delivery mechanisms will determine whether the benefits observed in clinical studies translate into meaningful reductions in RSV-related morbidity and mortality worldwide, particularly in low- and middle-income countries where the disease burden remains greatest.

Taken together, the emerging evidence suggests that the assessment of Nirsevimab is increasingly moving beyond demonstrating efficacy and safety in controlled clinical trials. Its expanding use in real-world settings has raised broader questions regarding the population-level consequences of widespread immunoprophylaxis, including potential changes in RSV epidemiology and viral evolution, the emergence and clinical relevance of escape variants, and its integration with maternal RSV vaccination. At the same time, economic sustainability, programmatic implementation, and equitable access are becoming increasingly important considerations. Future research should therefore adopt a broader perspective, addressing not only the direct protection afforded to individual infants but also the longer-term epidemiological, economic, and health-system implications of Nirsevimab implementation.

Several critical questions remain, including whether universal immunoprophylaxis will lead to lasting changes in RSV epidemiology, how protection will perform over successive seasons, how maternal and infant prevention strategies should be optimally combined, and whether emerging antibody technologies can maintain effectiveness in the context of viral evolution. Addressing these challenges will guide future RSV prevention policies and ensure that advances in immunoprophylaxis provide durable, accessible, and equitable protection for infants globally.

## 13. Conclusions

The advent of Nirsevimab has fundamentally reshaped the landscape of RSV prevention. As evidence continues to accumulate, its long-term success will depend not only on sustained clinical effectiveness but also on equitable access, successful implementation, and continued surveillance. However, important challenges remain regarding the durability of protection, the potential for viral evolution and the emergence of resistance-associated variants, the cost-effectiveness of Nirsevimab across different healthcare systems, and the optimal integration of Nirsevimab with maternal RSV vaccination. Together with maternal RSV vaccination, Nirsevimab marks the beginning of a new era in RSV prevention, with the potential to substantially reduce the burden of severe RSV disease and improve infant health on a global scale.

## Figures and Tables

**Figure 1:**
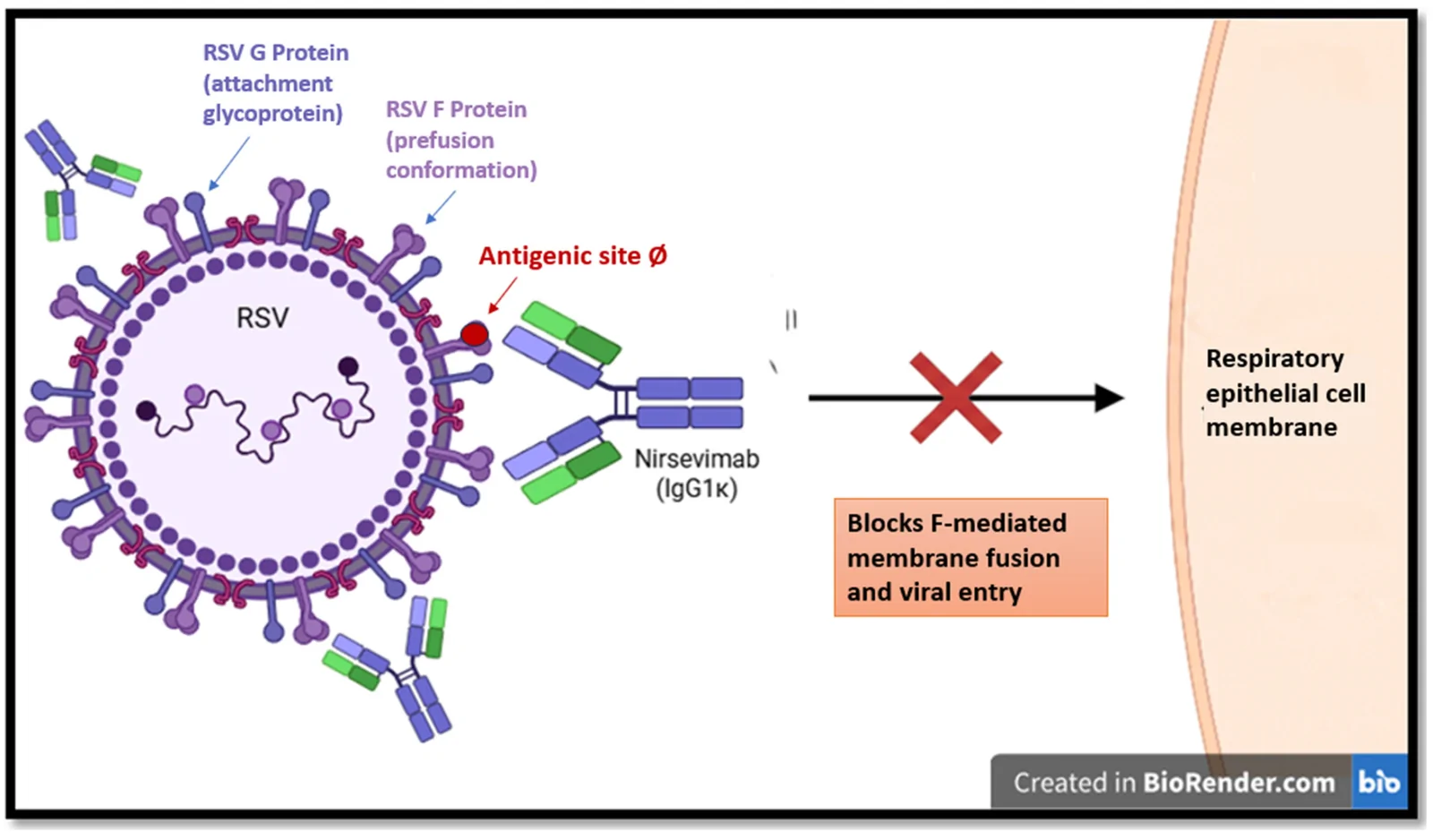
Molecular target and neutralization mechanism of Nirsevimab. Nirsevimab is a fully human recombinant IgG1κ monoclonal antibody that specifically binds antigenic site Ø on the prefusion conformation of the RSV fusion (F) protein. By targeting this highly conserved and vulnerable epitope, Nirsevimab stabilizes the prefusion F structure and prevents the conformational changes required for viral fusion with respiratory epithelial cells. Abbreviations: RSV, Respiratory syncytial virus; site Ø, site zero; IgG1k, human immunoglobulin G1 kappa; RSV F protein, Respiratory syncytial virus fusion protein. Created with Biorender (web-based version; BioRender.com, Toronto, ON, Canada).

**Figure 2:**
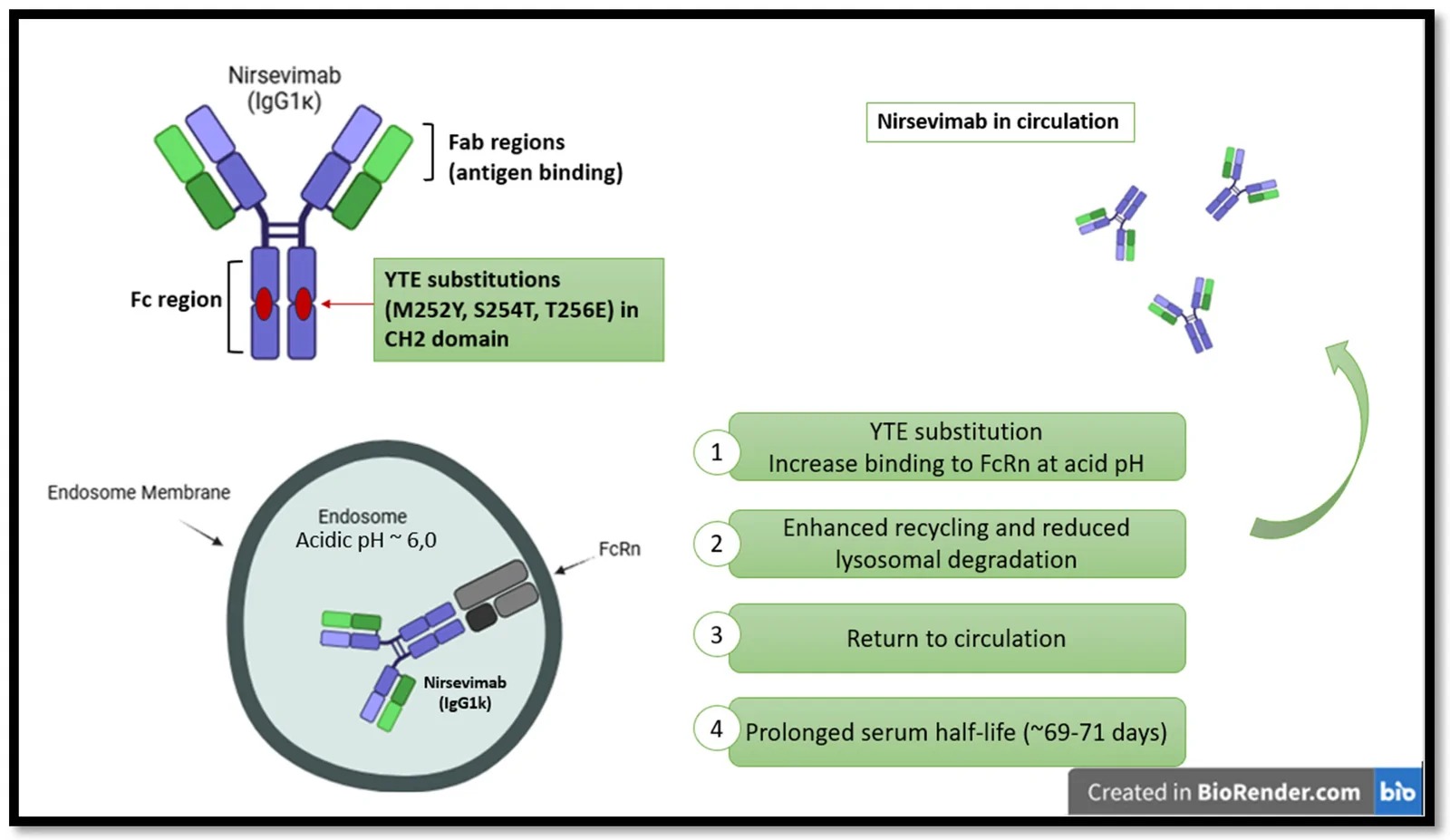
YTE Fc modification enhances the pharmacokinetic profile of Nirsevimab. The YTE (M252Y/S254T/T256E) Fc modification increases binding affinity to FcRn, facilitating antibody recycling and reducing degradation. This molecular optimization markedly prolongs the serum half-life of Nirsevimab, allowing season-long RSV protection following a single dose. Abbreviations: IgG1k, human immunoglobulin G1 kappa; FcRn, neonatal Fc receptor; Fc, fragment crystallizable region; Fab, fragment antigen-binding region; YTE, IgG1 Fc variant containing the M252Y, S254T, and T256E substitutions. Created with BioRender.

**Table 1:** Comparison of the main characteristics of Nirsevimab and palivizumab for RSV prevention.

Characteristic	Nirsevimab	Palivizumab
Antibody	Fully human recombinant IgG1κ	Humanized IgG1
Target	Antigenic site Ø of the prefusion RSV F protein	Antigenic site II of the RSV F protein
Half-life	~69–71 days	~20–30 days
Administration	Single intramuscular dose	Monthly intramuscular doses (up to 5 per RSV season)
Duration of protection	One RSV season (~5 months)	One month per dose
Eligible population	All infants in the first RSV season; selected high-risk children in the second season	Infants and young children at high risk for severe RSV disease

Abbreviations: RSV, Respiratory syncytial virus; IgG1κ, human immunoglobulin G1 kappa.

**Table 2:** Pivotal clinical trials evaluating the efficacy, safety, and pharmacokinetic profile of Nirsevimab in different infant populations.

Trial	Study Population	Study Design	Key Findings
**Phase 2b (NCT02878330)** [21]	Healthy preterm infants (29–34 + 6 weeks’ gestation)	Phase 2b, randomized, placebo-controlled	70.1% efficacy against medically attended RSV-LRTI and 78.4% against RSV-associated hospitalization.
**MELODY (NCT03979313)** [24]	Healthy term and late-preterm infants	Phase 3, randomized, placebo-controlled	74.5% efficacy against medically attended RSV-LRTI during the first RSV season.
**MEDLEY (NCT03959488)** [30]	Infants eligible for palivizumab (prematurity, CHD, CLD)	Phase 2/3, randomized, active-controlled	Comparable efficacy, safety, and pharmacokinetic profile to palivizumab, supporting use in high-risk infants.
**HARMONIE (NCT05437510)** [31]	Infants entering their first RSV season	Phase 3b, pragmatic, randomized	83.2% effectiveness against RSV-associated hospitalization and 75.7% against very severe RSV-LRTI.
Pooled Phase 2b + MELODY analysis [34]	Preterm and term infants	Prespecified pooled analysis	79.5% reduction in medically attended RSV-LRTI, 77.3% reduction in RSV hospitalization, and 86.0% reduction in very severe RSV disease.

Abbreviations: CHD, congenital heart disease; CLD, chronic lung disease; RSV, respiratory syncytial virus; LRTI, lower respiratory tract infection.

**Table 3:** Real-world effectiveness of Nirsevimab in the prevention of RSV-associated disease following implementation in routine clinical practice.

Study (First Author, Year)	Country	Study Design	Population (N)	Main Outcome	Key Findings
Payne et al., 2025 [35]	United States	Multicenter test-negative study	5039 infants	RSV-related ED visits and hospitalization	Nirsevimab effectiveness was 77% against RSV-related emergency department visits and 98% against RSV-associated hospitalization during the 2023–2024 RSV season.
Lefferts et al., 2024 [36]	United States (Alaska)	Observational study	472 infants	Medically attended RSV illness and RSV hospitalization	76% effectiveness against medically attended RSV illness and 89% effectiveness against RSV-related hospitalization in infants during their first RSV season
Torres et al., 2025 [37]	Chile	Retrospective observational study	154,173 infants	RSV hospitalization and ICU admission	Effectiveness reached 76.41% RSV-related LRTI hospitalizations, 84.94% RSV-related ICU admissions, 66.5% all-cause LRTI hospitalizations, and 47.9% of all-cause hospitalizations.
Barbas del Buey et al., 2024 [40]	Spain	Prospective population-based cohort study	33,859 infants	RSV hospitalization and ICU admission	Protection against RSV hospitalization was 93.6% at 30 days and 87.6% at 150 days, while effectiveness against ICU admission was 94.4% at 30 days and 92.1% at 90 days.
Chauvel et al., 2024 [38]	France	Retrospective observational study	83 infants	RSV-associated hospitalization	Effectiveness was 78.3% against RSV-associated LRTI hospitalization. RSV hospitalization incidence was reduced by 55% compared with pre-COVID-19 seasons and by 47% compared with the 2022–2023 season.
Fortunato et al., 2025 [39]	Italy	Population-based observational cohort study	4820 infants	RSV-associated hospitalization	Demonstrated 84.4% effectiveness against RSV-related LRTI hospitalization (screening method), 72.5% adjusted effectiveness using the test-negative design, and an adjusted relative risk reduction of 81.6%.

Abbreviations: RSV, respiratory syncytial virus; LRTI, lower respiratory tract infection; ED, emergency department; ICU, intensive care unit; COVID-19, coronavirus disease 2019.

## Data Availability

No new data were created or analyzed in this study.

## Abbreviations

The following abbreviations are used in this manuscript:

RSV	Respiratory syncytial virus
LRTI	Lower respiratory tract infection
MeSH	Medical Subject Headings
FDA	U.S. Food and Drug Administration
FcRn	Neonatal Fc receptor
IgG1κ	Human recombinant monoclonal antibody
MHRA	UK Medicines and Healthcare Products Regulatory Agency
ACIP	Advisory Committee on Immunization Practices
AAP	American Academy of Pediatrics
IM	Intramuscular injection
NPIs	Non-pharmaceutical interventions
ICU	Intensive care unit
VAERD	Vaccine-associated enhanced respiratory disease
RASs	Resistance-associated substitutions
QALY	Quality-adjusted life year
ICER	Incremental cost-effectiveness ratio
LMICs	Low- and middle-income countries
mAbs	Long-acting monoclonal antibodies
COVID-19	Coronavirus disease 2019
ED	Emergency department
